# DNA annealing by Redβ is insufficient for homologous recombination and the additional requirements involve intra- and inter-molecular interactions

**DOI:** 10.1038/srep34525

**Published:** 2016-10-06

**Authors:** Sivaraman Subramaniam, Axel Erler, Jun Fu, Andrea Kranz, Jing Tang, Mohanraj Gopalswamy, Saminathan Ramakrishnan, Adrian Keller, Guido Grundmeier, Daniel Müller, Michael Sattler, A. Francis Stewart

**Affiliations:** 1 Genomics, Biotechnology Center, TU Dresden, Tatzberg 47/49, 01307 Dresden, Germany; 2 Shandong University–Helmholtz Joint Institute of Biotechnology, State Key Laboratory of Microbial Technology, Shandong University, Shanda Nanlu 27, 250100 Jinan, People’s Republic of China; 3 Institute of Structural Biology, Helmholtz Zentrum München, 85764 Neuherberg, Germany and Center for Integrated Protein Science Munich (CIPSM), Department of Chemistry, Technische Universität München, Lichtenbergstr.4, 85747 Garching, Germany; 4 Technical and Macromolecular Chemistry, University of Paderborn, Warburger Str. 100 33098 Paderborn, Germany; 5 Department of Biosystems Science and Engineering (D-BSSE), ETH Zürich, Mattenstraße 26, 4058 Basel, Switzerland

## Abstract

Single strand annealing proteins (SSAPs) like Redβ initiate homologous recombination by annealing complementary DNA strands. We show that C-terminally truncated Redβ, whilst still able to promote annealing and nucleoprotein filament formation, is unable to mediate homologous recombination. Mutations of the C-terminal domain were evaluated using both single- and double stranded (ss and ds) substrates in recombination assays. Mutations of critical amino acids affected either dsDNA recombination or both ssDNA and dsDNA recombination indicating two separable functions, one of which is critical for dsDNA recombination and the second for recombination *per se*. As evaluated by co-immunoprecipitation experiments, the dsDNA recombination function relates to the Redα-Redβ protein-protein interaction, which requires not only contacts in the C-terminal domain but also a region near the N-terminus. Because the nucleoprotein filament formed with C-terminally truncated Redβ has altered properties, the second C-terminal function could be due to an interaction required for functional filaments. Alternatively the second C-terminal function could indicate a requirement for a Redβ-host factor interaction. These data further advance the model for Red recombination and the proposition that Redβ and RAD52 SSAPs share ancestral and mechanistic roots.

In living cells the genome is constantly being damaged by environmental agents, oxidative stress and replication errors^1^. Amongst an arsenal of repair pathways, double strand break repair initiated by DNA annealing proteins is central to the maintenance of genomic integrity^2^,^3^. By mechanism, DNA annealing proteins are divided into ATPases capable of strand invasion (RecA, RAD51) and single strand annealing proteins (SSAPs) that do not utilize ATP^4^. Until recently SSAPs were classified into at least three separate groups each named after the most prominent member; RAD52, Redβ and Erf^5^. Of these, RAD52 is best understood because it is highly conserved in all eukaryotes and involved in cancer mechanisms^6^,^7^,^8^,^9^. Redβ from λ phage is also notable because it mediates the very useful DNA engineering technology termed recombineering^10^,^11^,^12^. In 2009, using deep bioinformatic tools to identify a distant signature motif, we proposed that RAD52 and Redβ are members of an SSAP superfamily^13^. This proposition has been supported by the application of different bioinformatic methods^14^,^15^.

As opposed to single strand (ss) DNA binding proteins such as SSB and RPA, which protect and occlude ssDNA from recombination, RAD52/Redβ SSAPs promote recombination and share several biochemical similarities. They are weak ssDNA binding proteins with no affinity for double-stranded (ds) DNA. In the absence of DNA *in vitro*, they multimerize into rings or chains at high concentrations (>0.5 μM). Also, they share a similar protein architecture based on an N-terminal ssDNA binding domain of ~180 amino acids and a C-terminal extension that in the case of RAD52 is required for homologous recombination (HR) through specific protein-protein interactions^6^. These shared biochemical, protein sequence and functional similarities suggest the existence of an ancestral annealing mechanism involved in HR.

RAD52 is the best characterized SSAP due, in part, to two crystal structures of the N-terminal DNA binding domain of human RAD52^16^,^17^. Both crystal structures revealed that the N-terminal domain of ~200 amino acids forms a mushroom-shaped undecameric ring with an external groove lined with positive charges, which probably binds the phosphodiester backbone of ssDNA. Although full length RAD52 forms a heptameric, not undecameric, multimer^18^, a RAD52 homolog from a *Lactococcus* phage, SakRad52, also forms undecameric rings without DNA *in vitro*^19^. Along with undecameric/dodecameric rings formed by Redβ^20^, the recurrence of these beautiful ~11 mer rings shared amongst various SSAPs has promoted ring-based models for homology searching and DNA annealing^17^,^20^,^21^,^22^,^23^. The ring models have been challenged by our recent experiments, which indicate that Redβ homology searching occurs by a monomer to monomer random hit mechanism at concentrations below the *in vitro* threshold concentration for formation of the ~11 mer rings^13^,^24^.

Whether mediated by monomers or rings, four lines of evidence indicate that annealing by Redβ initiates recombination on the lagging strand template at the replication fork. The first indication arose from strand bias observed using ss oligonucleotides (oligos). The ss oligos that can act as Okazaki-like primers for lagging strand synthesis consistently delivered more recombination than their complementary oligos^25^,^26^. Second, Red recombination requires ongoing replication at the moment of recombination and not merely to amplify the recombination product^27^. Third, dsDNA substrates are processed into full length ssDNA intermediates by Redα before annealing by Redβ into the replication fork^27^,^28^. Fourth, host mutations that enlarge the ssDNA loop on the lagging strand template at the replication fork increase the frequency of Red recombination^29^.

In addition to roles in genome maintenance, SSAPs are also found in phages and viruses with a 5′ to 3′ exonuclease as “SynExo” pairs^30^. The Red (recombination deficient) operon in λ phage is a SynExo paradigm pairing Redα, a 5′ to 3′ exonuclease that is a toroidal homotrimer^31^,^32^, with Redβ. The Redα/Redβ SynExo pair interacts through a specific protein-protein interaction^33^ that is required for efficient homologous recombination using ds^34^ but not ss^25^ DNA insertions. To date, neither the molecular detail nor the function of this Redα/Redβ protein-protein interaction has been defined, partly due to the lack of a Redβ crystal or NMR structure.

Here we further characterize the Redα-Redβ protein-protein interaction and examine Redβ structure and function. This information is integrated with existing data into a new model for concerted action by Redα and Redβ.

## Results

### Both N- and C-terminae of Redβ are essential for recombination

**Figure 1:**
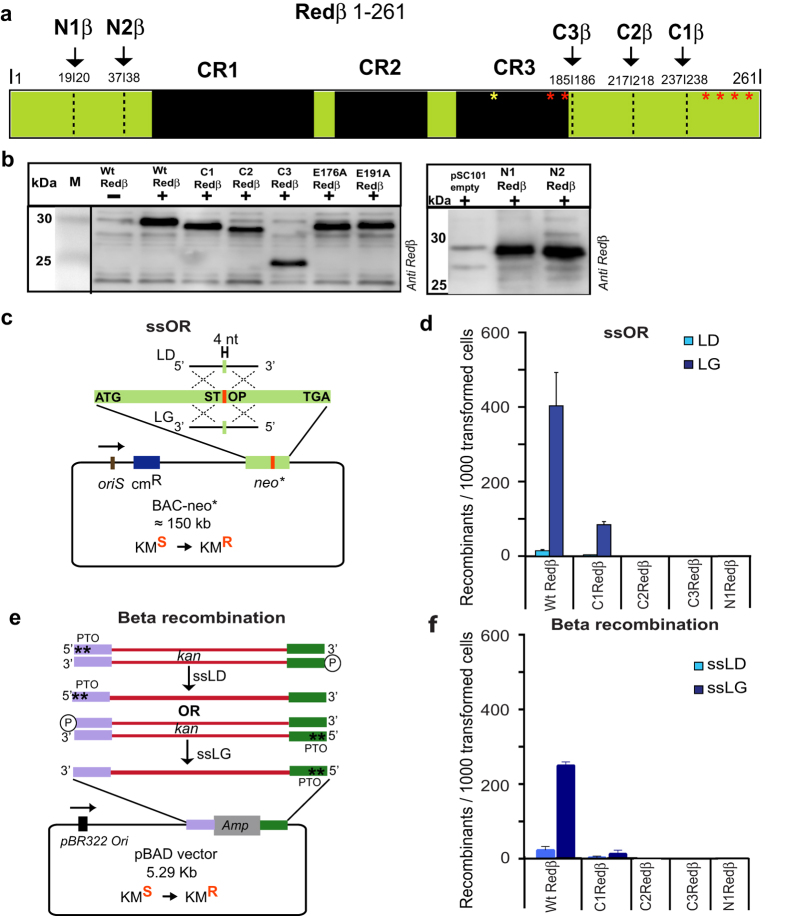
Redβ requires both N- and C-terminae for homologous recombination. (**a**) Diagram of Redβ depicting the CR1–3 regions conserved with other phage SSAPs^5^ as well as the deletions and point mutations used in this work. (**b**) Expression of Redβ from pSC101BAD-Redβ induced by arabinose and evaluated by Western using a Redβ antibody. Lane Wt Redβ-; uninduced with arabinose: lane pSC101 empty; induced with arabinose but without Redβ in the expression plasmid. (**c**) Schematic representation of the ssOR (single strand Oligonucleotide Repair) assay. The neomycin resistance gene was mutated to introduce a central stop codon (neo*) and inserted into a BAC. Repair of the mutation by incorporation of an oligonucleotide restores kanamycin resistance. The complementary single strand oligonucleotides can either anneal to the lagging strand template and act as an Okazaki-like fragment primer (LG) or not (LD). The BAC resides in an *E. coli* host, which is induced for expression of wt and mutant Red proteins from a pSC101 plasmid by addition of arabinose 45 minutes before electroporation of the oligonucleotide followed by quantitating the acquisition of kanamycin resistance by counting colonies on kanamycin plates. (**d**) Performance of wt and truncated Redβ proteins in the ssOR assay using either the LD oligo (light blue) or LG oligo (dark blue). Experiments were performed in triplicates and data were normalized to the number of cells transformed with a control plasmid and are represented as mean ± SD. (**e**) Schematic representation of the Beta recombination assay. Two PCR products were generated to carry the neo (kanamycin resistance) gene. Both products carried identical DNA sequence however differed according to the position of two consecutive phosphothioate bonds at opposing 5′-ends. In both PCR products, the other 5′ end was phosphorylated. After electroporation, only one strand will be digested by Redα into full length single strands as illustrated. The strand whose 3′ end can serve as a primer for Okazaki-like fragment synthesis is termed ssLG and the other strand is termed ssLD. The kanamycin resistance gene (kan) is flanked by 50 bp homology arms (purple and green) that flank the ampicillin resistance gene in pBAD. (**f**) Performance of the wt and truncated Redβ proteins in the Beta recombination assay. Experiments were performed as described in (**d**).

Redβ encompasses three parts; a central region that is defined by its conservation with other SSAPs^5^ flanked by N- and C-terminal regions of 47 and 83 amino acids respectively (Fig. 1a). The conserved region is required for both DNA binding and annealing whereas the C-terminal region is dispensable for annealing^15^,^35^ and its function remains undefined. Whether the N-terminus is required for DNA binding, annealing or recombination has not been determined. To further dissect Redβ function, we generated two N-terminal truncations, N1Redβ (20–261) and N2Redβ (38–261) and three C-terminal truncations, C1Redβ (1–237), C2Redβ (1–217) and C3Redβ (1–185). All deletions were well expressed as evaluated by Western blotting (Fig. 1b; Supplementary Fig. 1a). Functional testing for recombination activity in *E. coli* was evaluated using either a single strand oligonucleotide repair (ssOR) assay in a BAC (bacterial artificial chromosome; Fig. 1c) or a Beta recombination assay^36^ based on a dsDNA substrate with one 5′ end protected against exonuclease digestion by a pair of phosphothioate bonds (Fig. 1e). All Redβ truncations disabled recombination in both assays (Fig. 1d,f; data not shown) except for the least C-terminally truncated construct C1Redβ (1–237), which retained approximately 25% of the wt level in the ssOR assay as well as the expected bias between lagging and leading strands. These results indicate that the recombination functions of Redβ rely on amino acids that lie outside of the conserved region at both ends of the protein and the very C-terminus is required for dsDNA but not ssDNA recombination.

### DNA annealing is not sufficient for *in vivo* recombination

**Figure 2:**
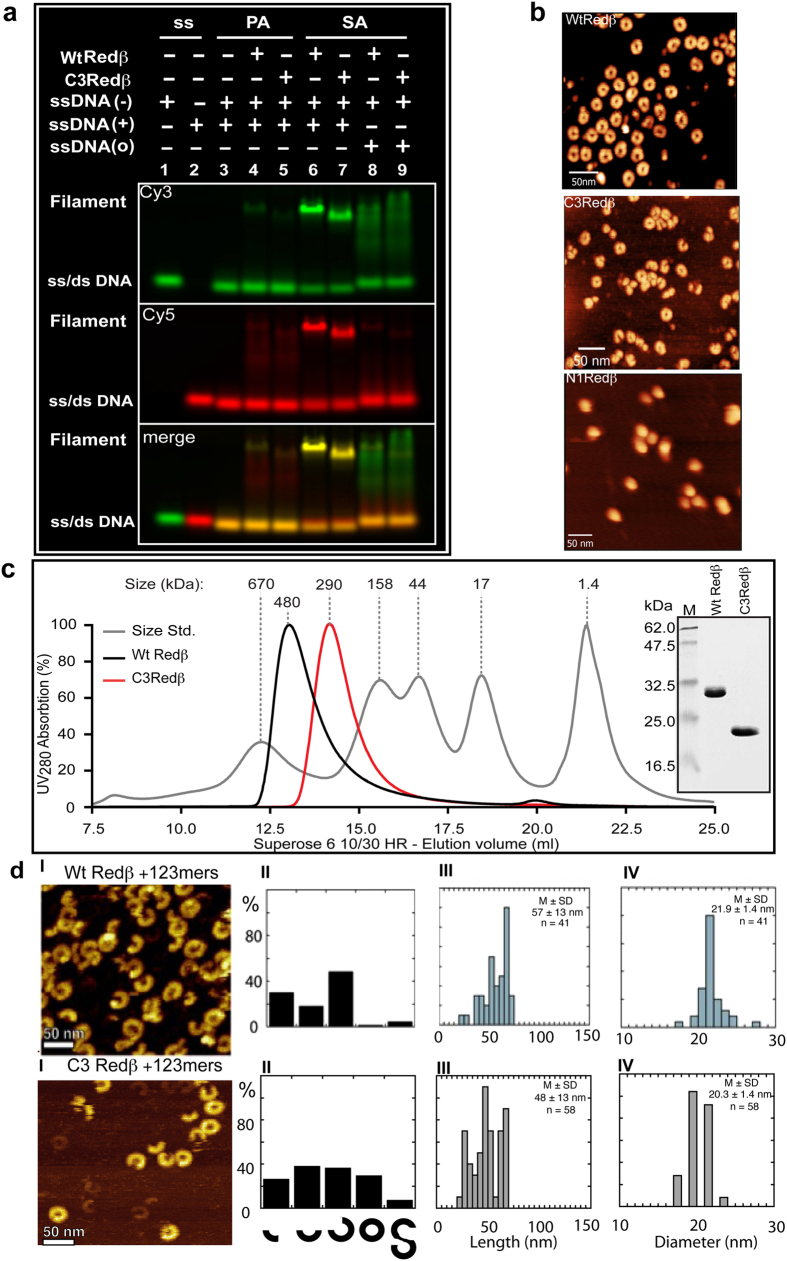
C3Redβ forms rings and can anneal DNA to form the nucleoprotein filament. (**a**) Electrophoretic shift mobility assay (EMSA) for Redβ-DNA complexes (wt Redβ and C3Redβ) using Cy3/Cy5 labeled complementary 50 nt ssDNA strands. All lanes have the Cy3 labeled oligo (−) except lane 2. All lanes have the Cy5 labeled complementary oligo (+) except lanes 1, 8 and 9. Lanes 8 and 9 have a non-complementary Cy5 labeled oligo (o). PA; pre-annealed (lanes 3–5) – the complementary oligos were pre-annealed before adding the indicated proteins. SA; sequentially added (lanes 6–9) – the Cy3 labeled oligo was incubated with the indicated protein before addition of the Cy5 labeled oligo. The presence of a faint band shift and slight red smear in lanes 4 and 5 indicates that the oligo preannealing did not go to completion, which Redβ completed (green band) and there was excess Cy5 oligo (red smear). The annealed nucleoprotein filament complex can be seen in lane 6 (wt Redβ) and the C3-truncation (lane 7). In lanes 8 and 9, the smear shows the weak ssDNA binding of both wt and C3-truncated Redβ to the Cy3 labeled oligo. Oligonucleotide sequences are shown in Suppl. Fig. 5. (**b**) Atomic Force Microscopy (AFM) images of wt Redβ, C3Redβ and N1Redβ imaged without DNA. To achieve a reasonable distribution of adherent complexes on the mica surface, input concentrations of 0.7, 2.0 and 2.6 μM respectively were used. (**c**) Analytical size exclusion chromatography of purified wt Redβ (black line) and C3Redβ (red line) on a Superose 6 column. The grey curve shows the molecular standards with respective sizes. Inset: Coomassie-stained SDS-PAGE gel (15%) showing purified recombinant C-terminal StrepII tagged wt Redβ and C3Redβ proteins. (**d**) AFM images of wt Redβ and C3Redβ. Panels I: representative images of the filaments formed by wt Redβ (upper) and C3Redβ (lower) annealing two complementary 123 mers; Panels II: histograms of the different filament species illustrated below. Panels III: histograms of filament length (nm). Panels IV: histograms of filament diameters (nm). M, mean; SD, standard deviation; n, number of particles analyzed.

To determine whether the Redβ deletion mutants lost recombination because they lost the ability to anneal DNA, we purified N1Redβ and C3Redβ. The wild type and two mutant proteins were all well expressed and soluble with similar secondary structural properties as evaluated by circular dichroism (Supplementary Fig. 1b). Then we evaluated their annealing capacities by gel shift. N1Redβ failed to promote annealing (Supplementary Fig. 1c,d), which provides a straightforward explanation for the lack of recombination and also indicates that the annealing domain includes the poorly conserved sequences at the N-terminus. In contrast, the biggest C-terminal deletion, C3Redβ, showed qualitatively similar DNA binding and annealing properties as wt Redβ, including weak ssDNA binding and formation of the annealed nucleoprotein filament upon sequential addition of complementary strands (Fig. 2a). These observations confirm previous work with a C-terminal truncation (Redβ 1–177), which demonstrated normal annealing activity upon sequential addition of complementary oligonucleotides^35^.

### Quaternary structures of Redβ truncations

In the absence of DNA, wt Redβ has been shown to form rings^20^ and/or a shallow right-handed open helix equated to a ‘split-lock washer’^13^. Using atomic force microscopy, we observed that C3Redβ retained this property whereas N1Redβ appeared as balls rather than rings (Fig. 2b).

Wt Redβ, C3Redβ and N1Redβ were compared by size exclusion chromatography (SEC) at an input concentration of 350 μM (1 mg/ml). Redβ eluted as a single peak at ~480 kDa apparent size (Fig. 2c), which is greater than calculated for a complex of 11 to 12 monomers (11/12 × 29 = ~335 kDa) but consistent with the expectation that these rings/shallow helices should produce a larger Stokes radius than predicted by their molecular mass. C3Redβ eluted as a single peak at ~290 kDa again larger than expected for 11 to 12 monomers (11/12 × 20 = ~230 kDa) with approximately the same proportional discrepancy as wt Redβ (480/335 = 1.4; 290/230 = 1.3). Additionally the half-maximum peak widths of wt Redβ and C3Redβ were almost identical suggesting comparable oligomeric states and degrees of heterogeneity. However N1Redβ eluted at an apparently larger size than wt Redβ (Supplementary Fig. 1e), which is consistent with its altered appearance in AFM.

To extend this analysis, we examined the SEC peaks after elution using dynamic and static light scattering (DLS, SLS; Table 1). Wt Redβ presented two well separated species one corresponding to the monomer (Species 3) and the other to the apparent 11/12-mer complex (Species 1; Table 1). This observation indicates that wt Redβ dissociates from the multimeric complex upon dilution. In contrast, for both C3Redβ and N1Redβ, less than 10% of the populations were apparently monomeric and the samples were more broadly dispersed than wt Redβ displaying additional intermediate-sized multimers (Table 1). Notably N1Redβ showed the least propensity to dissociate. These observations indicate that both the N- and C-terminae modulate Redβ intra-molecular interactions in the absence of DNA by potentially destabilizing the multimeric forms. Removal of either the N- or C-terminae apparently alters and stabilizes the homomeric interactions.

### AFM comparison of the wt and truncated Redβ nucleoprotein filaments

Wt Redβ and C3Redβ were analyzed by AFM imaging after annealing two 123 mer oligonucleotides as described previously^13^. The images revealed that C3Redβ also forms left-handed nucleoprotein helical filaments like Redβ, however distinct differences were apparent (Fig. 2d). The Redβ filaments were quite uniform in presentation, length and diameter, whereas the C3Redβ filaments were more heterogeneous and shorter with smaller diameters (Fig. 2d), again indicating that whereas the C-terminus is not essential for formation of the nucleoprotein filament, it modulates filament properties.

### Point mutations at Redβ C-terminus that selectively debilitate beta recombination

**Figure 3:**
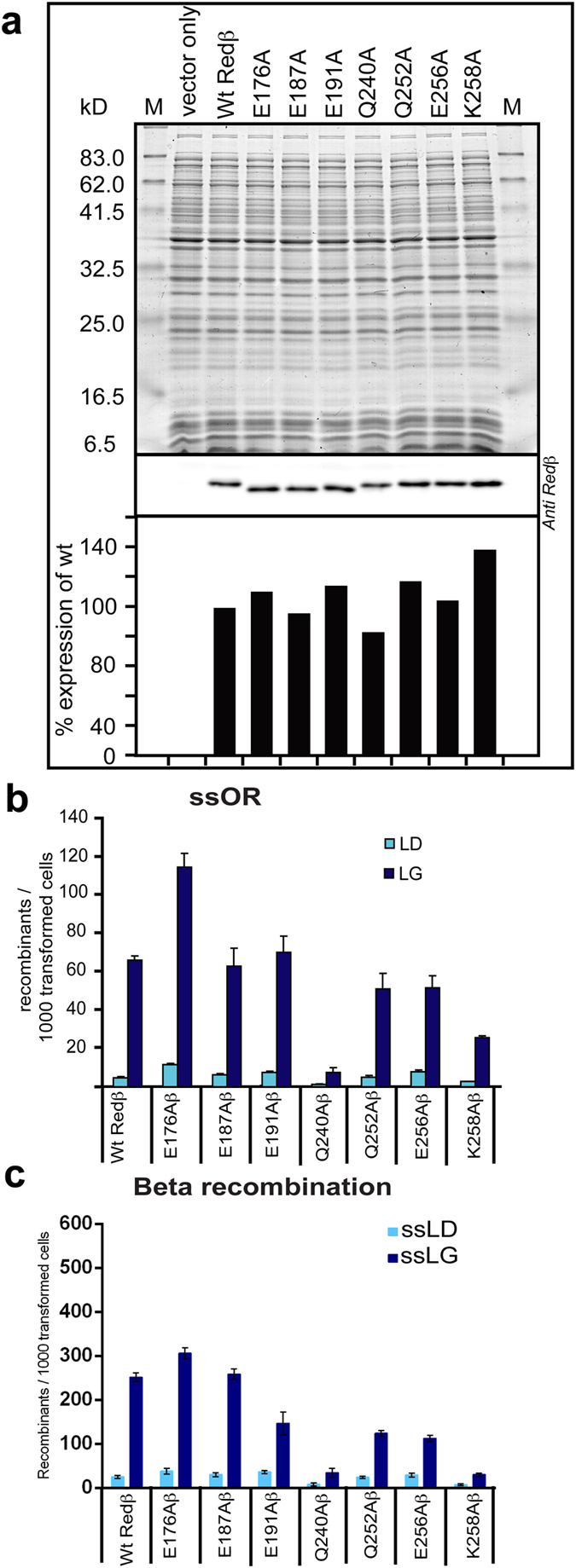
Point mutations of Redβ C-terminus show reduced recombination efficiencies. (**a**) Western blot analysis showing equivalent protein expression levels of the seven Redβ point mutants. The upper panel shows the 12% SDS-PAGE stained with Coomassie Brilliant Blue above the Western probed with an anti-Redβ antibody, which is quantified below (wt Redβ = 100%). (**b**) The indicated Redβ point mutations, expressed from pSC101-BAD-Redβ plasmids, were evaluated using the ssOR assay described in Fig. 1c,d. (**c**) The indicated Redβ point mutations, expressed from pSC101-BAD-Redγβα plasmids, were evaluated using the Beta recombination assay described in Fig. 1e,f.

To investigate the properties of the Redβ C-terminus in more detail, we created a series of alanine point mutations in the terminal C1 deletion region and tested them in the ssOR and Beta recombination assays (Fig. 3). As controls we employed alanine point mutations around the C-terminal end of the annealing domain (E176A, E187A, E191A). All point mutations were similarly expressed under recombination conditions (Fig. 3a). Mutations E176A and E187A did not impair recombination in either assay whereas Q240A strongly reduced it in both. In contrast, like the C1Redβ deletion (Fig. 1d,f), mutations E191A, Q252A, E256A and K258A affected beta recombination more than ssOR. Indeed K258A and C1Redβ presented a similar profile suggesting that the phenotype of the C1 deletion is largely due to the removal of this lysine. Also notable was the fact that all debilitating mutations either reduced both ds and ss recombination or mainly reduced ds recombination alone. These results indicate that although the C-terminus is not required for annealing, its contribution to recombination can be partially separated into (i) a function(s) required for both ds and ss recombination and (ii) a function(s) required for ds but not ss recombination.

### Redβ interactions with Redα

**Figure 4:**
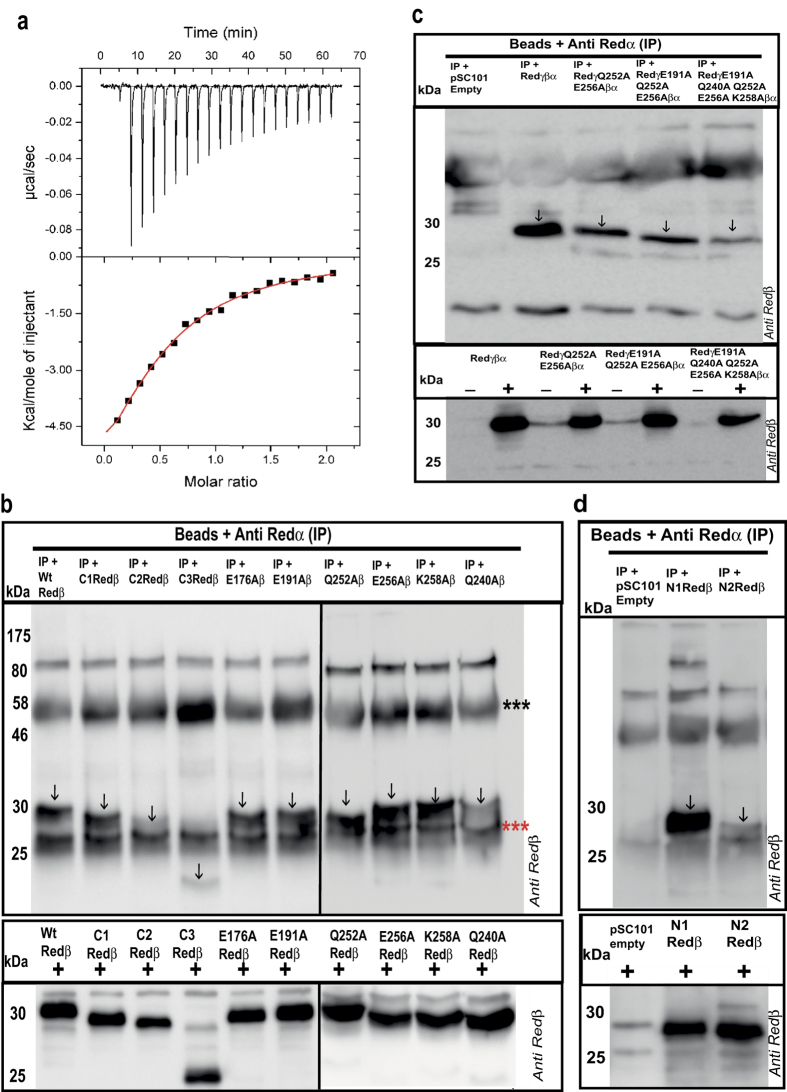
Evaluation of the Redα-Redβ interaction. (**a**) Isothermal titration calorimetry determined by injecting Redα (2 μl; 150 μM; 3 minute intervals) into the cell containing Redβ (15 μM) and the resulting heat signal is plotted against time (*upper panel*) with the binding isotherm plotted in the lower panel including the theoretical fit to a single class of binding site (red line). (**b**) Upper panel: Co-IP of Redβ mutations using anti-Redα antibody. The Western blots of the immunoprecipitates were probed with anti-Redβ antibody. Vertical arrows indicate the Redβ protein. The three red and black asterisks indicate the immunoglobulin light and heavy chains respectively. Lower panel: Western blots showing the expression of different Redβ protein truncations and point mutants, which were used as input for the Co-IPs. (**c**) As for (**b**) except the left most lane on the upper panel shows a Co-IP from cells containing pSC101 without any inserted Red genes; and the lower panel shows Redβ protein expression without (−) and with (+) arabinose induction of Red protein expression. (**d**) As for (**b**,**c**). In accordance with journal policy, uncropped versions of these Western blots are presented in Supplementary Fig. 4.

Recombination initiated by the Red proteins can utilize either dsDNA or ssDNA substrates. For recombination with dsDNA, the specific protein-protein interaction between Redα and Redβ^33^ is important^34^. However recombination using ssDNA oligonucleotides does not require Redα^25^,^26^. Consequently impaired Redα-Redβ protein-protein interaction is a likely explanation for the C-terminal mutations that impaired ds recombination more than ssOR. To test this idea, we first determined the equilibrium dissociation constant for the Redα-Redβ protein-protein interaction using isothermal titration calorimetry (ITC; Fig. 4a). Redα (150 μM) was injected into a cell containing 15 μM Redβ or buffer with injection volumes of 2 μl at 3 minute intervals. A K_D_ of 7.9 +/− 0.8 μM was determined. Next we performed co-immunoprecipitation experiments using Redα and Redβ antibodies with good properties (Fig. 4b–d; Supplementary Figs 2 and 3). Immunoprecipitations by the Redα antibody of Redα interacting with the C2 and C3 deletions were impaired but the C1Redβ deletion and all the single point mutations tested, except Q240A, were efficiently immunoprecipitated. Assuming that single point mutations may not be sufficient to debilitate the protein-protein interaction in a biochemical assay, we made several combinations and found that Q252A/E256A, E191A/Q252A/E256A and E191A/Q240A/Q252A/E256A/K258A indeed showed decreased Redα interaction (Fig. 4c). Also notable is the observation that the interaction was not abolished in any of the mutant proteins but only weakened. Consistent with this, co-immunoprecipitation of N-terminal deletions revealed an interaction site between amino acids 20 and 38, whose deletion again reduced but did not abolish the interaction (Fig. 4d).

### Neo-tail assay as a tool to decipher the mechanism of Redαβ interactions

**Figure 5:**
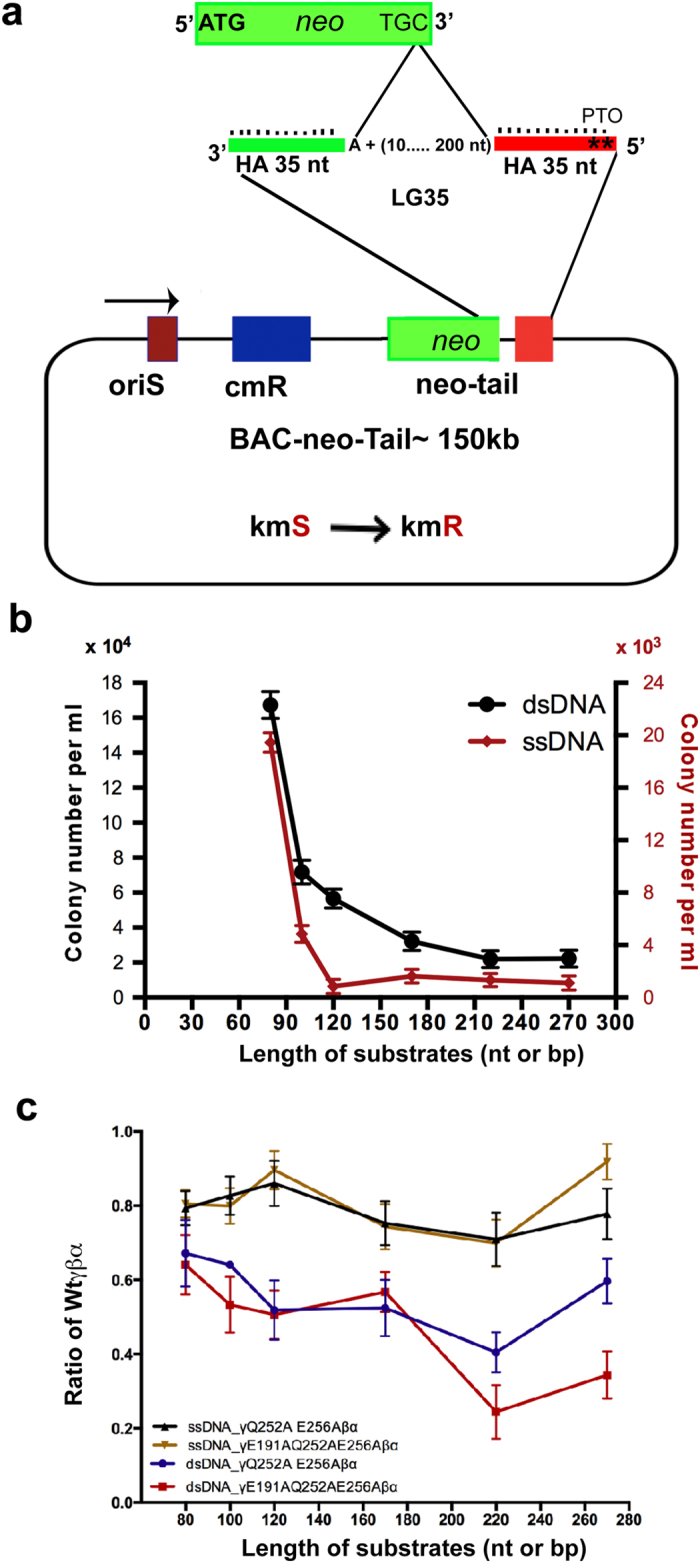
Redβ C-terminal point mutations that decrease the Redα interaction impair Beta recombination but not ssOR. (**a**) Schematic representation of the *neo*-tail assay. The stop codon of the neo gene was mutated to TGC. Homologous recombination to revert the stop codon to TGA will restore kanamycin resistance. Six dsDNA substrates were prepared, each including the same 35 bp homology arms (green and orange) and a pair of phosphothioate bonds at the 5′ end of the strand whose 3′ end can serve to prime Okazaki-like fragment synthesis. The six substrates differed by the lengths between the homology arms (10 + 70 = 80, 30 + 70 = 100, 50 + 70 = 120, 100 + 70 = 170, 150 + 70 = 220 and 200 + 70 = 270 bp) and were electroporated as either ds or ssDNA after *in vitro* Redα digestion. (**b**) *Neo*-tail assay comparison of the six substrates as ds (black) or ss (red) DNA in homologous recombination mediated by wild type Redβ. Redβ was either expressed from pSC101-BAD-Redβ (ss) or pSC101-BAD-Redγβα (ds) and the results are from three independent experiments each performed in triplicate. Data were normalized to the number of cells transformed with a control plasmid and are represented as mean ± SD. (**c**) Recombination efficiencies for Redβ Q252A, E256A and Redβ E191A, Q252A, E256A expressed as a ratio compared to wt Redβ using the *neo*-tail assay with ss and dsDNA substrates. Error bars indicate the mean ± SD of an experiment performed in triplicate.

To test the functional significance of the Redα-Redβ interaction, we devised a new assay termed “neo-tail”, which exploits the fact that the neomycin phosphotransferase protein does not tolerate any additional amino acids at its C-terminus^37^. We mutated the stop codon of the neomycin phosphotransferase gene so that it does not convey kanamycin resistance (Fig. 5a). The stop codon and consequently kanamycin resistance is restored by recombination using ssDNA or dsDNA substrates that all share exactly the same 5′ and 3′ homology arms even when they are separated by different length inserts. Using the same homology arms to restore the same antibiotic resistance reduces the number of variables in the assay thereby permitting a very accurate evaluation of increasing insert length on recombination efficiency. Using the neo-tail assay and 35 nt/bp 5′ and 3′ homology arms, we evaluated a range of inserts from 10 to 200 corresponding to 80 (35 + 10 + 35) to 270 (35 + 200 + 35) nucleotide or base pair substrates. The substrates were prepared for electroporation as either 5′ phosphothioated ssDNA or asymmetrically 5′ phosphothioated dsDNA (Supplementary Fig. 4). In both cases, the recombinogenic ssDNAs, either the provided ssDNAs or the Redα product after digestion of the dsDNAs *in vivo*, are identical and the homology arm at the 3′ end will serve as an Okazaki fragment primer when annealed to the lagging strand template.

To characterize the neo-tail assay, we compared the ssDNA and dsDNA substrates in recombination mediated by wild type Redαβγ, which revealed two differences (Fig. 5b). First, overall recombination with ssDNA substrates was at least an order of magnitude less efficient than with dsDNA substrates. Second, recombination with ssDNA showed a precipitous drop in efficiency at lengths above the 80 nucleotide substrate and all substrates longer than 100 were equally inefficient. Recombination efficiency with dsDNA also fell with increasing lengths, however the decrease was more uniform.

We attribute these two differences to the action of Redα with the following model. For dsDNA substrates, Redα and Redβ act in concert through their specific protein-protein interaction that loads Redβ onto the ssDNA emerging from Redα exonuclease digestion. This processive loading of Redβ not only promotes recombination by preventing collapse of the ssDNA onto itself but also may facilitate the exploration for complementary sequences by Redβ bound ssDNA. These advantages are not available when ssDNA substrates are provided. In particular, the folding of the ssDNA upon itself occludes Redβ binding thereby reducing recombination. In this experiment, the 80 mer ssDNA appears to be reasonably accessible for Redβ binding whereas longer lengths adopted occluding conformations.

To test this interpretation and to evaluate functionally the Redα-Redβ protein-protein interactions identified in Fig. 4, we compared the Redβ point mutation combinations Q252A/E256A, E191A/Q252A/E256A and E191A/Q240A/Q252A/E256A/K258A with wt Redβ in the same assay. Consistent with the Q240A mutation, E191A/Q240A/Q252A/E256A/K258A mutations inactivated Redβ (data not shown). The other two point mutation combinations had a mild effect on ssDNA recombination regardless of substrate length whereas they had a stronger impact on dsDNA recombination (Fig. 5c) indicating that the contribution of the Redα-Redβ protein-protein interaction to recombination had been impaired by the mutations.

## Discussion

Due to similar biochemical and physical properties, including weak ssDNA but no dsDNA binding affinity, multimerization to form rings and a similar protein architecture based on an N-terminal DNA annealing domain with C-terminal extension, we proposed that Redβ and RAD52 are members of an ancestrally related SSAP superfamily. Here we add further evidence to support the proposition that SSAPs share an ancestral and functional relationship. Like RAD52^6^, Redβ includes a poorly conserved N-terminus that is nevertheless required for annealing as well as protein-protein interactions C-terminal to the DNA annealing domain that are required for recombination.

In a search for the role of the C-terminal domain in Redβ recombination, three issues arose involving (i) the protein-protein interaction between Redα and Redβ; (ii) an additional activity required for both ds and ssDNA recombination and (iii) an intrinsic contribution to Redβ multimerization. These three will be discussed in turn.

Because they co-purify, the protein-protein interaction between Redα and Redβ, has been known for at least 30 years^33^. However the functional relevance of this interaction has not been deeply investigated. In a search for a way to use homologous recombination in *E. coli* to engineer recombinant DNA, we discovered the very useful properties of the SynExo pairs, RecE/RecT from rac phage and Redα/Redβ from λ phage^10^,^11^,^38^, which initiated recombineering technology^12^,^39^ and also prompted the question: can these related phage SynExo pairs swap partners? That is, can the RecE exonuclease co-operate with Redβ SSAP and vice versa, can the Redα exonuclease co-operate with RecT SSAP? The answer was conclusively negative. The heterotypic combinations do not mediate homologous recombination between dsDNA substrates suggesting the protein-protein interactions are specific and required for function. Supporting this suggestion, we showed that RecE and RecT, like Redα and Redβ, also specifically interact^34^.

Hence the requirement for the C-terminal domain in dsDNA recombination could be readily explained if the Redα interaction lies in this region. Mutagenesis of the C-terminal domain provided support for this explanation. Several mutations (C1; E191A; Q252A; E256A: K258A) impaired dsDNA recombination more than ssDNA recombination. However, in co-IP experiments, none of these mutations alone impaired the Redα-Redβ interaction. Potentially these data indicate a discrepancy between the mutagenic impact of the single mutations on recombination *in vivo* compared to the biochemical protein-protein interaction. This discrepancy may be due to the relative insensitivity of co-IP experiments compared to functional recombination assays. However it may also be due to the complexity of the protein-protein interaction. In particular we discovered that the protein-protein interaction also depends upon amino acids at the N-terminus between residues 20 and 37. Also selected combinations (Q252A/E256A; E191A/Q252A/E256A) both impaired the protein-protein interaction and debilitated dsDNA recombination more than ssDNA recombination. These findings indicate that either Redβ is folded to juxtapose amino acids 20–37, 191 and 252–261 to compose the Redα interaction surface or that the Redα-Redβ interaction has a dynamic component potentially employing different aspects of the interaction during ongoing recombination. We favor the latter possibility because it lends an explanation for the role of the Redα-Redβ interaction in dsDNA recombination. A dynamic component in the protein-protein interaction is also indicated by the relatively modest affinity (K_D_ = 7.9 μM), which is sufficient to provide specificity but weak enough for easy disassembly.

The identification of Redα interaction with the C-terminal domain does not explain why ssDNA recombination also requires the C-terminal domain. In this regard, the Q240A mutation is notable because it debilitates both ss and dsDNA recombination. This mutation slightly impairs expression (Fig. 3a) so possibly perturbs folding of the C-terminal domain. Alternatively the drastic mutagenic impact on both ss and dsDNA recombination may indicate the existence of another function required for recombination, such as a protein-protein interaction with a host factor. Our recent experience exporting Red recombination into other prokaryotes suggests that Red-host protein-protein interactions are significant^40^,^41^. The proposition that the C-terminal domain not only interacts with Redα but also another factor required for recombination is also supported by the C1 deletion, which debilitates recombination (Fig. 1d,f) but does not reduce the interaction with Redα (Fig. 4a).

A third contribution from the C-terminal domain involves its effect on multimerisation, annealing and formation of the nucleoprotein filament. Both in the absence of DNA and upon annealing, the properties of the multimeric forms of truncated Redβ differed from wt Redβ, as did the balance between monomeric and multimeric forms. The ability of the C-terminal domain to modulate the multimeric properties of the DNA binding domain may have a functional impact on recombination, possibly by tuning the balance between monomeric and multimeric forms to optimize the homology search. However, further studies are required to evaluate the merits of this proposition. Notably, our observations on the Redβ C-terminal domain are concordant with the recent publication by Smith and Bell^42^. Deletion of the N-terminus leads to similar deductions. Like the C3 deletion, the N1 deletion showed a greatly reduced propensity to dissociate to monomers (Table 1). Additionally as visualized by AFM, the multimeric forms of the N1 deletion without DNA are balls rather than rings (Fig. 2a). This suggests that although the N1 deletion retains part of the specific homomeric interaction, the N-terminus is required for both an orderly interaction and the instability of this interaction.

**Figure 6:**
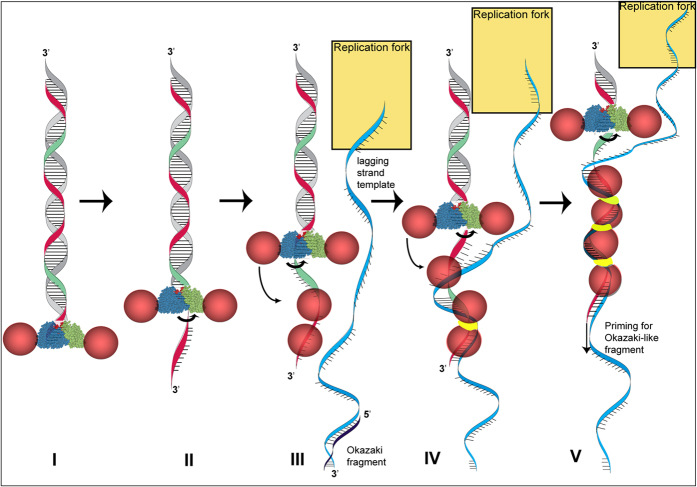
Model for Redαβ molecular crosstalk. (**I**) Redα toroids (blue and green cones) thread onto the 3′ end of a dsDNA break (**I**) and digest nucleotides from the 5′ end, extruding the 3′ end as single stranded (red strand; (**II**)). Redβ monomers (pink spheres) bound to Redα are promoted to bind to the emerging ssDNA (**III**). Upon locating complementary sequence on the lagging strand template (blue strand) at the replication fork, a Redβ dimer undergoes a conformational change (yellow band) to establish a DNA clamp (**IV**) that nucleates nucleoprotein filament growth and allows, possibly promotes, the 3′ end (red strand) to serve as a primer for Okazaki-like fragment synthesis (**V**). The replication fork is depicted as a golden rectangle.

Previously we unraveled the mechanism of Red-mediated dsDNA recombination, which occurs on the lagging strand template at the replication fork, to explain a part of its remarkable efficiency and usefulness^27^. Here we address other aspects related to Redβ recombination functions and the role of the protein-protein interaction with Redα. Combining the data presented here with previous knowledge permits the following model for dsDNA recombination (Fig. 6). Red recombination begins with Redα exonuclease digestion. Redα is a homotrimeric toroid, which loads onto the 3′ end and removes nucleotides from the 5′ end of the complementary strand whilst extruding the 3′ ended single strand through the center of the toroid^31^,^32^. Redα binds Redβ at 1:1 stoichiometry^33^. We propose that Redα promotes Redβ loading onto the emerging ssDNA before it can fold onto itself and occlude Redβ binding. Redβ binds weakly to ssDNA and promotes annealing^43^ by random collision promoted by bound monomers to search for candidate sequence matches, which are converted into very stable dimeric Redβ DNA clamps upon successful identification of complementarity^13^,^24^. These clamps nucleate the zipping-up of complementary strands by growth of a Redβ nucleoprotein filament. This process occurs at the replication fork as its progression exposes single stranded DNA regions predominantly on the lagging strand template ahead of Okazaki fragment synthesis. Once annealed into the replication fork, the 3′ end of the incoming DNA strand serves as an Okazaki-like replication primer for lagging strand synthesis. Then a mutation or recombinant strand will be incorporated if Redβ has also annealed complementary sequences from the 5′ end of the incoming ssDNA to the lagging strand template^27^,^28^,^29^.

## Methods

All experiments were performed in *E. coli* strain GB2005 (F-mcrA Δ(mrr-hsdRMS-mcrBC) φ80lacZΔM15 ΔlacX74 recA1 endA1 araD139 Δ (ara, leu) 7697 galU galK l rpsL nupG fhuA::IS2 recET redα, phage T1-resistent). For *in vivo* assays, Redα, Redβ, N1Redβ (20–261), C1Redβ (1–237), C2Redβ (1–217) and C3Redβ (1–185) were inserted into pSC101, which depends on the temperature sensitive oriR101^44^, and expressed from the L-arabinose inducible pBAD promoter^45^.

### Recombination assays

For the beta recombination assay, the dsDNA was prepared by PCR amplification using the Phusion HF-DNA polymerase with one primer carrying two phosphothioate (PTO) bonds at the 5′ end and the other primer having a 5′ phosphate as described^27^. PCR amplified dsDNA was purified using Invisorb Fragment CleanUp (Stratec Molecular GmbH) and 100 pmol was electroporated for each data point. For the neo-tail assay, the Redγβα operon (accession codes: NP_040616, NP_040617 and NP_040618) was similarly cloned in pSC101. The point mutations RedβE176A, RedβE187A, RedβE191A, RedβQ240A, RedβQ252A, RedβE256A and RedβK258A and combinatorial point mutants (RedγβQ252A, E256Aα), (RedγβE191A, Q252A, E256Aα) and (RedγβE191A, Q240A, Q252A, E256A, K258Aα) were made using synthesized gene segments (GeneArt, Life Technologies) and linear plus linear recombineering with RecE/RecT^46^. All mutations were confirmed by sequencing. The insert size series (10, 30, 50, 100, 150 and 200 bp) was cloned in p15A. The inserts plus 35 bp flanking homology arms were PCR amplified and purified as described above for the beta recombination assay. To generate ssDNA, the PCR products were digested with Redα (purified from pASK expression) and the reaction was cleaned up using ssDNA/RNA clean-up concentrator kit (Zymo Research, USA). The purified dsDNA and ssDNA were checked by gel analysis (data not shown). 100 pmol of either dsDNA or ssDNA were used for electroporation. For counting colonies to estimate recombination efficiency, the low number of colonies obtained from the empty pSC101 control was subtracted from all other values in the same experiment.

### Protein expression

For *in vitro* studies, Redα and SSAP proteins were produced using one step Strep-tag II/Strep-Tactin affinity chromatography, which allows mild protein purification under physiological conditions^47^. Initially, the ORFs of Redα and Redβ were cloned individually into pASK-IBA2 (IBA Göttingen, Germany) under transcriptional control of the tetracycline promoter^48^. The eight amino acid long Strep-tag II (WSHPQFEK) was fused to the Redα C-terminus and Redβ N-terminus separated from the respective end by a Ser-Ala spacer. Overexpression of the tagged proteins and purification was described previously^13^.

### Affinity and preparative gel filtration chromatography

The cleared lysate was loaded onto a gravity flow Strep-Tactin Superflow Sepharose column with a bed volume of 4.0 ml, which was equilibrated with Buffer W. Washing steps were carried out extensively for 40 column volumes. The recombinant protein was eluted by addition of the Strep-tag II specific competitor D-desthiobiotin to Buffer W at a final concentration of 2.5 mM. Recombinant protein containing fractions were pooled, dialyzed against storage buffer (25 mM Tris-HCl, pH 8.0, 50 mM NaCl, 1 mM DTT, 0.1 mM EDTA, 30% (v/v) glycerol) in a small volume using disposable dialysis tubes of the Mini Dialysis Kit 8 kDa cut-off (GE Healthcare), concentrated by ultrafiltration using Vivaspin 6 ml concentrators with twin vertical membranes (Vivascience) and the protein concentration was determined using either Bradford protein reagent (Sigma) or UV quantification. Quality and quantities of the purification process were monitored by analytical SDS-PAGE analysis. Purified Strep-tag II tagged proteins were generally more than 95% pure at a concentration range of 0.5–10.0 mg/ml.

To obtain highly homogenous protein preparations, the affinity chromatography eluates were injected in to Superdex 200 column (column volume 120 ml) connected to an ÄKTAexplorer 10S system (GE Healthcare). The column was equilibrated with 50 mM Tris-HCl pH 8.0, 150 mM NaCl, 1 mM MgCl_2_. The flow rate was kept constant at 1.0 ml/min all through the procedure. The eluent protein concentration was constantly monitored by spectroscopic UV detection at 280 nm. Desired peaks were pooled, concentrated and quantified on a Nanodrop 1000 (Thermoscientific GmbH).

### Electrophoretic mobility shift assays

Protein-DNA complexes were generated by incubation of 10 μM protein with 1 μM DNA in Tris-buffer (20 mM Tris-HCl, pH 7.5, 10 mM NaCl, 10 mM MgCl_2_) at 37 °C for 30 min, followed by addition of the complementary oligo and incubation for a further 30 minutes (when relevant). Samples were then mixed with 10x orange gel loading dye (Li-Cor Biosciences) and separated on 1.2 to 1.6% agarose gels in 2x TBE buffer. DNA was fluorescently-labelled by ordering oligonucleotides with 5′-fluorescent Cy3 or Cy5 dyes. Fluorescent signals were detected and recorded using Typhoon 9410 Variable Mode Imager (GE Healthcare).

### Analytical size exclusion chromatography

Purified Strep-Tag II C-terminally fused Redα and SSAP proteins were run in high-resolution Superose 6 10/30 HR column (GE Healthcare) under native solution conditions. An exclusion limit of 4000 kDa was used, with an optimal separation range from 5 to 500 kDa and a bed volume of 24 ml was connected to an ÄKTAexplorer 10S system (GE Healthcare). Column equilibrations, size calibrations and sample analysis were performed in 25 mM Hepes pH 8.0, 150 mM NaCl, 1 mM MgCl_2_ and 0.5 mM DTT buffer at a flow rate of 0.3 ml/min and a backpressure of 1.2 MPa. The column was calibrated using a gel filtration standard (Bio-Rad), composed of five molecular weight markers namely thyroglobulin (670 kDa), bovine gamma-globulin (158 kDa), chicken ovalbumin (44 kDa), equine myoglobin (17 kDa) and vitamin B12 (1.35 kDa). Protein samples of 1.0 mg were loaded into a 100 μl injection loop and subsequently injected onto the column. The eluent protein concentration was constantly monitored by spectroscopic UV detection at 280 nm. For reproducibility each protein was injected at least two times using a 100 μl injection loop.

### Size exclusion chromatography (SEC) combined with dynamic light scattering (DLS) and static light scattering (SLS)

To determine apparent molecular weight of different Redβ preparations, we employed batch light scattering applications using DLS and SLS/MALS (WYATT, USA) with a Dawn Heleos II 18-angle-detector in combination with SEC. We performed the SEC using Sephadex200 column (GE Life) plugged to HPLC system with autosampler (Agilent), dRI, UV and SLS/MALS and injected purified Redβ proteins at 1 mg/ml concentration. Every injection was repeated twice and analysed using Astra 5 (Wyatt) for molar mass distribution calculations.

### Atomic Force Microscopy (AFM) imaging and data analysis

AFM Imaging was done as described previously^13^ except for the AFM images of Fig. 2b (wt Redβ, C3Redβ, N1Redβ), which were recorded dry using an Agilent 5100 AFM and HQ:NSC18/Al BS cantilevers (MikroMasch) in intermittent contact mode. All data analysis and data fitting was done in IGOR Pro version 5.04B (WaveMetrics Inc.). The refined models (Fig. 2D) were generated employing computer-aided design (CAD) construction software SolidWorks 2005 SPO.0 (SolidWorks Corporation) and by the support of Quindium GmbH & Co. KG.

### Isothermal titration calorimetry

ITC experiments were performed at 25 °C on an iTC200 (GE Healthcare). Redα and Redβ were dialyzed overnight in buffer containing 20 mM HEPES, 150 mM sodium chloride, 1 mM magnesium chloride, 0.5 mM TECP, pH 8, using 5000 MWCO Spectra/Por DispoLyzers (Spectrum Laboratories, Rancho Dominguez, CA). 150 μM Redα was injected into the cell containing 15 μM Redβ. An automatic injection syringe was used with injection volumes of 2 μl at 3 min intervals, rotating at 750 rpm. Data acquisition and subsequent nonlinear regression analysis were performed using ORIGIN software supplied with the instrument. Experimental data were fitted to a single-site binding model to extract binding stoichiometry (n), the dissociation constant (K_D_) and the enthalpy change (ΔH) as previously described^49^. Thermodynamic parameters were calculated from the Gibbs free energy equation, ΔG = ΔH − TΔS = −RT ln (1/Kd), where ΔG and ΔS are the changes in free energy and entropy of binding, respectively and ΔG was −7.0 kcal/mol. ΔH = −9.84 +/− 1.12 kcal/mol; −TΔS = 2.87 kcal/mol (ΔS = −9.64 cal/mol/deg). T is the absolute temperature and R = 1.9 l/mol/K. Control experiment were performed by titrating Redα in buffer and corrected for the heat of dilution.

### Immunoprecipitation and co-immunoprecipitation

GB2005 carrying pSC101 encoding Redγβα with or without various mutations in Redβ was freshly diluted from an overnight culture in LB media plus 3 μg/ml tetracycline and grown at +30 °C until OD600 ~0.4. Protein expression was then induced with L-arabinose 0.3% for 45 minutes at +37 °C. Cells were cooled on ice for 5 minutes and centrifuged at 13,200 rpm for 1 minute at +4 °C. Following washing two times with ice cold 1xPBS, cells were resuspended in the lysis buffer (100 mM Tris-HCl pH 8.0; 150 mM NaCl and 1 mM EDTA) supplemented with complete bacterial protease inhibitor cocktail (Sigma) and sonicated using a sonicating water bath (Diagenode GmbH) with 30 second on and off cycles for 5 minutes. The cell debris was removed by centrifugation at 16, 100 g, 30′, 4 °C.

Immunoprecipitation reactions used 50–60 μl protein-G sepharose beads (GE Healthcare) in 20% ethanol per 1.5 ml Eppendorf tube. The beads were washed twice with 1 ml of 1xPBS followed by centrifugation at 0.8 g for 30 seconds at room temperature. Following this, beads were washed 3 times in 1 ml of immunoprecipitation buffer (IP buffer) comprising (50 mM Tris-Cl pH 8.0, 150 mM NaCl, 10% glycerol, 1% triton X-100) supplemented with complete bacterial protease inhibitors cocktail (Sigma). Immunoprecipitation was initiated by adding 2 μl of anti-rabbit anti-Redα or anti-Redβ sera to the beads in 500 μl of IP buffer followed by immediate addition of the cell lysates from the protein expression. IP reactions were carried out at room temperature either for 20 minutes or 1 hour. Following the IP reaction, the beads were washed 4 times with 1 ml of IP buffer then suspended in SDS-PAGE Laemmli loading buffer, then incubated +95 °C for 5 minutes. After centrifugation, the supernatant was loaded on to a 12% reducing gel and electrophoresed at 130 V for 90 minutes before Western blotting. Immunoprecipitation reactions were confirmed by using anti-Redα antibody while the Co-IP reactions were monitored using anti-rabbit anti-Redβ antibody. The membranes were analyzed with chemiluminescent detection method using Luminata Forte Western HRP Substrate. The images were documented with Image quant LAS machine 4000 (GE Healthcare) and AIDA software, version 3.20 (Raytest).

## Additional Information

**How to cite this article**: Subramaniam, S. *et al*. DNA annealing by Redβ is insufficient for homologous recombination and the additional requirements involve intra- and inter-molecular interactions. *Sci. Rep*. **6**, 34525; doi: 10.1038/srep34525 (2016).

## Supplementary Material

Supplementary Information

## Figures and Tables

**Table 1:** Mass distributions for wt, C3 and N1Redβ after size exclusion chromatography.

Protein	Species 1				Species 2				Species 3			
	Rh (nm)	Dist (%)	~Mw (kDa)	No. of Subunits	Rh (nm)	Dist (%)	~Mw (kDa)	No. of Subunits	Rh (nm)	Dist (%)	~Mw (kDa)	No. of Subunits
WtRedβ	13.5 ± 4.4	31.8	490	11–12	—	—	—	—	4.5 ± 0.6	68.2	29 ± 4	1
C3Redβ	15.7 ± 3.6	45.1	520	12–13	8.3 ± 1.5	45.6	58	2–3	4.0 ± 1.4	9.3	26 ± 1	1
N1Redβ	15.1 ± 0.6	84.3	512	12–13	9.3 ± 0.6	8.3	56	2–3	3.9 ± 0.8	7.4	22 ± 3	1

The wt Redβ, C3Redβ and N1Redβ peaks fractionated by size exclusion chromatography (Fig. 2c), were analyzed by DLS and SLS, which revealed two (wt) and three (C1, N1) species as summarized with hydrodynamic radius (Rh) and distributions (Dist) as percentages together with estimated molecular weights and the corresponding number of Redβ molecules per species.
