# Supplementary material for: DNA annealing by Redβ is insufficient for homologous recombination and the additional requirements involve intra- and inter-molecular interactions
Source: Sci Rep. 2016 Oct 6;6:34525. doi: 10.1038/srep34525 (PMC5052646; doi:10.1038/srep34525)
Supplement: Supplementary Information [file srep34525-s1.pdf]

DNA annealing by Redβ is insufficient for homologous recombination and the additional requirements involve intra- and inter-molecular interactions

Sivaraman Subramaniam[1], Axel Erler[1], Jun Fu[1,2], Jing Tang[1], Mohanraj Gopalswamy[3], Saminathan Ramakrishnan[4], Adrian Keller[4], Guido Grundmeier[4], Daniel Müller[5], Michael Sattler[3] and A. Francis Stewart[1]

Supplementary Figure Legends

Supplementary Figure 1. (a) Quantification of protein expression from the Western blot analysis shown in Fig.1b. Wt Redβ was taken as 100%. **(b)** Far-UV CD spectrum of purified wt Redβ (blue), C3Redβ (maroon), and N1Redβ (orange) recorded at 10 μM (0.1 mg/ml) protein at 25 °C in 1x phosphate buffer with sodium fluoride. Proper folding and α-helical structure are indicated by the local minima at 208 and 222 nm. **(c)** and **(d)** Electrophoretic mobility shift assays (EMSAs) to study the single strand annealing properties of wt Redβ (c) and N1Redβ (d) using different lengths of ssDNA substrates from 10 to 22 nucleotides as indicated above the gels. To evaluate annealing, the proteins were pre-incubated with one strand for 10' at RT before the complementary strand was added 10' prior to electrophoresis. Concentrations: oligonucleotides 50pM; protein 10pM. Arrows indicate the nucleoprotein complex. **(e)** Analytical Size exclusion chromatography (SEC) shows the co-elution of N1Redβ along with the molecular standards (Bio-rad) on a Superose 6 column (GE healthcare). The X-axis indicates the elution volume (ml) and the Y-axis show the UV absorbance at 280nm as mAU. Inset: Coomassie-stained SDS-PAGE gel (15%) showing purified recombinant C-terminal StrepII tagged wt Redβ, N1Redβ and C3Redβ proteins.

Supplementary Figure 2. (a) Control western blot showing the input cell lysates expressing Redγβα probed with anti-Redα antibody. The cell lysates were uninduced (-) or induced (+) for protein expression from pSC101-BAD-Redγβα **(b)** Immunoprecipitation (IP) western using anti-Redα antibody of whole cell extract from cells uninduced (-) and induced (+) with arabinose for protein expression pSC101-BAD-Redγβα followed by western blot probed using both anti-Redα and anti-Redβ antibodies. The red asterisks show the IgG light chain and the black asterisks show the IgG heavy chain from anti-Redβ IP. **(c)** Co-immunoprecipitations (Co-IP) to define the Redα-Redβ interaction using anti-Redβ antibody on a 4-20% bis-Tris gel. Left panel: Co-IP for Redα probed with anti-Redα antibody. The input cell lysates expressing Redγβα from pSC101-BAD-Redγβα is shown. – and + indicates the cell lysates uninduced and induced for protein expression. The black arrow pointing downwards depicts the input Redα and co-migration of Redα with the light chain of Ig from anti-Redβ antibody. The red and black asterisks shows the light chain and heavy chain of Ig from anti- Redβ antibody respectively. Right panel: IP for Redβ probed with anti-Redβ antibody. The input cell lysates expressing Redγβα from pSC101-BAD-Redγβα is shown. – and + indicates the cell

lysates uninduced and induced for protein expression. The black arrow pointing downwards depicts the Redβ. The white arrow depicts the bait Redβ protein. The red and black asterisks show the light chain and heavy chain of Ig from anti-Redβ antibody respectively.

Supplementary Figure 3. Uncropped gel images. Co-immunoprecipitations (Co-IP) to define the Redα-Redβ interaction – uncropped blot pictures **(3 a1 top and 3 a2 top)** Co-IP of Redβ mutations using anti-Redα antibody. **(3 a1 top)** Uncropped Co-IP westerns from **Figure 4 b** showing the Co-IP results of wt Redβ, C1 Redβ, C2 Redβ, C3 Redβ, E176A Redβ, E191A Redβ. **(3 a2 top)** Uncropped Co-IP westerns from **Figure 4b** showing results of Q252A Redβ, E256A Redβ, K258A Redβ and Q240A Redβ. **(3 a1 bottom and 3 a2 bottom)** Uncropped western blots showing the expression of different Redβ protein truncations and point mutants, which were used as input for the Co-IPs. **(3 a1 bottom)** Western blot showing the expression of wt Redβ, C1 Redβ, C2 Redβ, C3 Redβ, E176A Redβ, E191A Redβ. – and + show the uninduced and induced conditions using arabinose for protein expression. **(3 a2 bottom)** Western blot showing the expression of Q252A Redβ, E256A Redβ, K258A Redβ and Q240A Redβ. – and + show the uninduced and induced conditions using arabinose for protein expression.

Co-immunoprecipitations (Co-IP) to define the Redα-Redβ interaction – uncropped blot pictures **(3 b top1)** Uncropped low exposure image of Co-IP westerns from **Figure 4c** showing the Co-IP results of wt Redγβα, RedγQ252A E256Aβα, RedγE191A Q252A E256Aβα and RedγE191A Q240A Q252A E256A K258Aβα. (**3 b top2)** Uncropped high exposure image of Co-IP westerns from **Figure 4c** showing the Co-IP results of wt Redγβα, RedγQ252A E256Aβα, RedγE191A Q252A E256Aβα and RedγE191A Q240A Q252A E256AK258Aβα. The number of exposures between the **3 b top1** and **3 b top2** is 15 with 10 seconds exposure time for each exposure. **(3 b bottom)** Uncropped western blot image showing the expression of Wt Redγβα, RedγQ252A E256Aβα, RedγE191A Q252A E256Aβα and RedγE191A Q240A Q252A E256A K258Aβα. - and + show the uninduced and induced conditions using arabinose for protein expression.

Co-immunoprecipitations (Co-IP) to define the Redα-Redβ interaction – uncropped blot pictures **(3 c top and 3 c bottom)** Co-IP of Redβ mutations using anti-Redα antibody. **(3 c top)** Uncropped Co-IP westerns from **Figure 3 d** showing the cell lysate induced (+) for Redα expression for showing the specificity of anti Redβ antibody and Co-IP results of vector only control (pSC101-Empty), Redα, N1Redβ, N2Redβ. **(3 c bottom)** Uncropped western blot image showing the expression of vector only control (pSC101-Empty), N1Redβ, N2Redβ. - and + show the uninduced and induced conditions using arabinose for protein expression.

Supplementary Figure 4. Quality control of the dsDNA and ssDNA substrate used in the neo-tail assay. **(a)** Agarose gel electrophoresis of linear dsDNA of different lengths generated by PCR. The lengths are marked above the corresponding lanes. Lanes 1 and 2 show the

100bp ladder and the Ultra low DNA ladder. **(b)** Native Urea-PAGE gel showing the pure ssDNA substrates of different lengths generated using Redα exonuclease *in vitro*. Each lane is marked with the respective length of the ssDNA loaded.

Supplementary Figure 1.

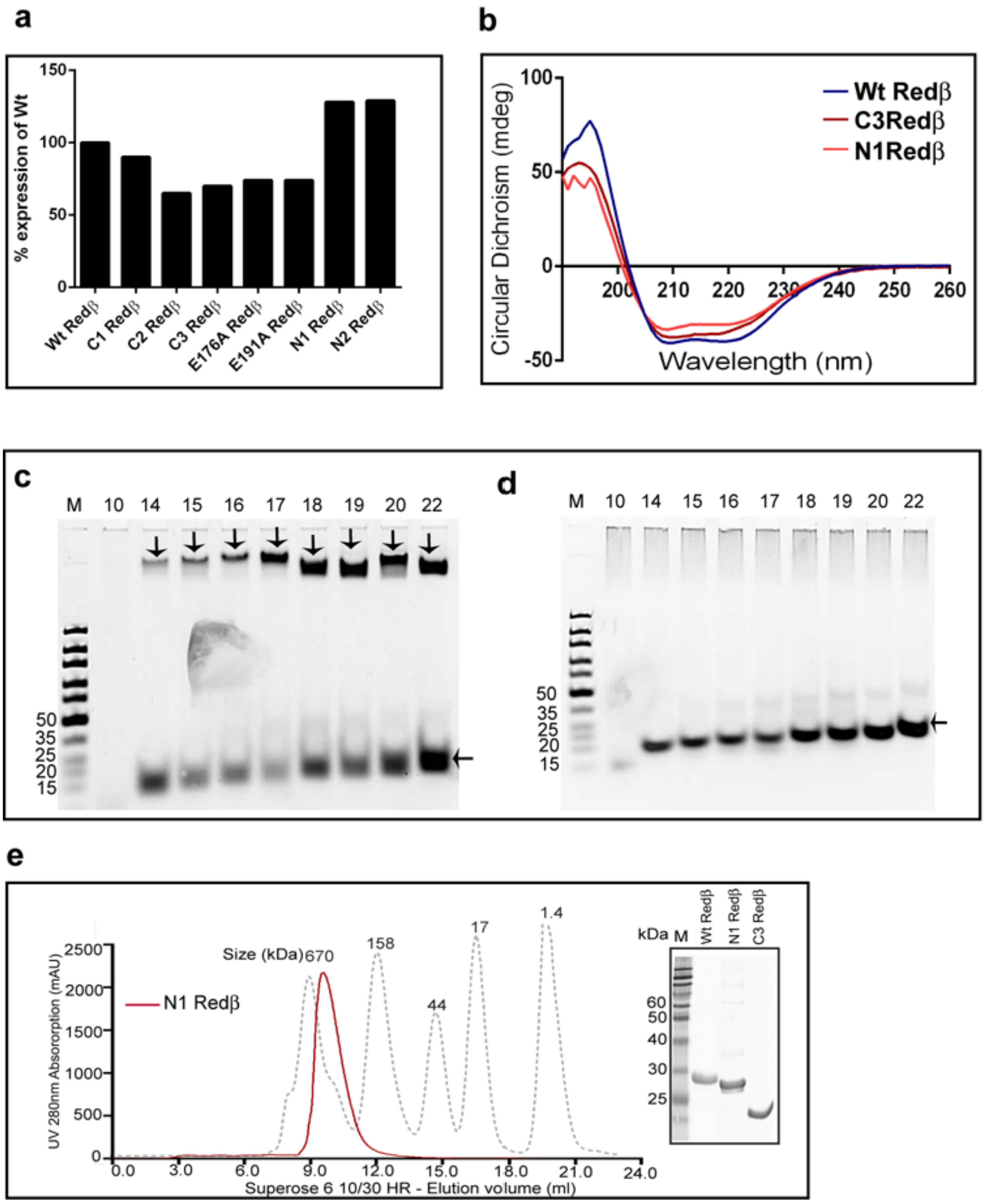

Supplementary Figure 2.

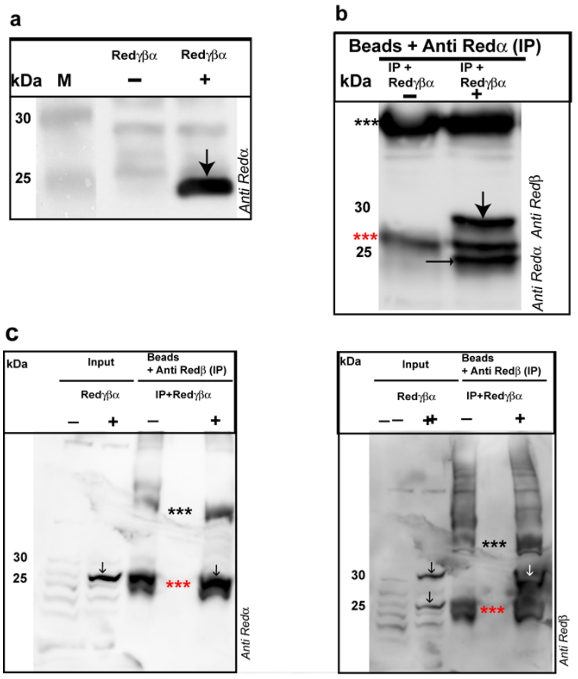

Supplementary Figure 3.

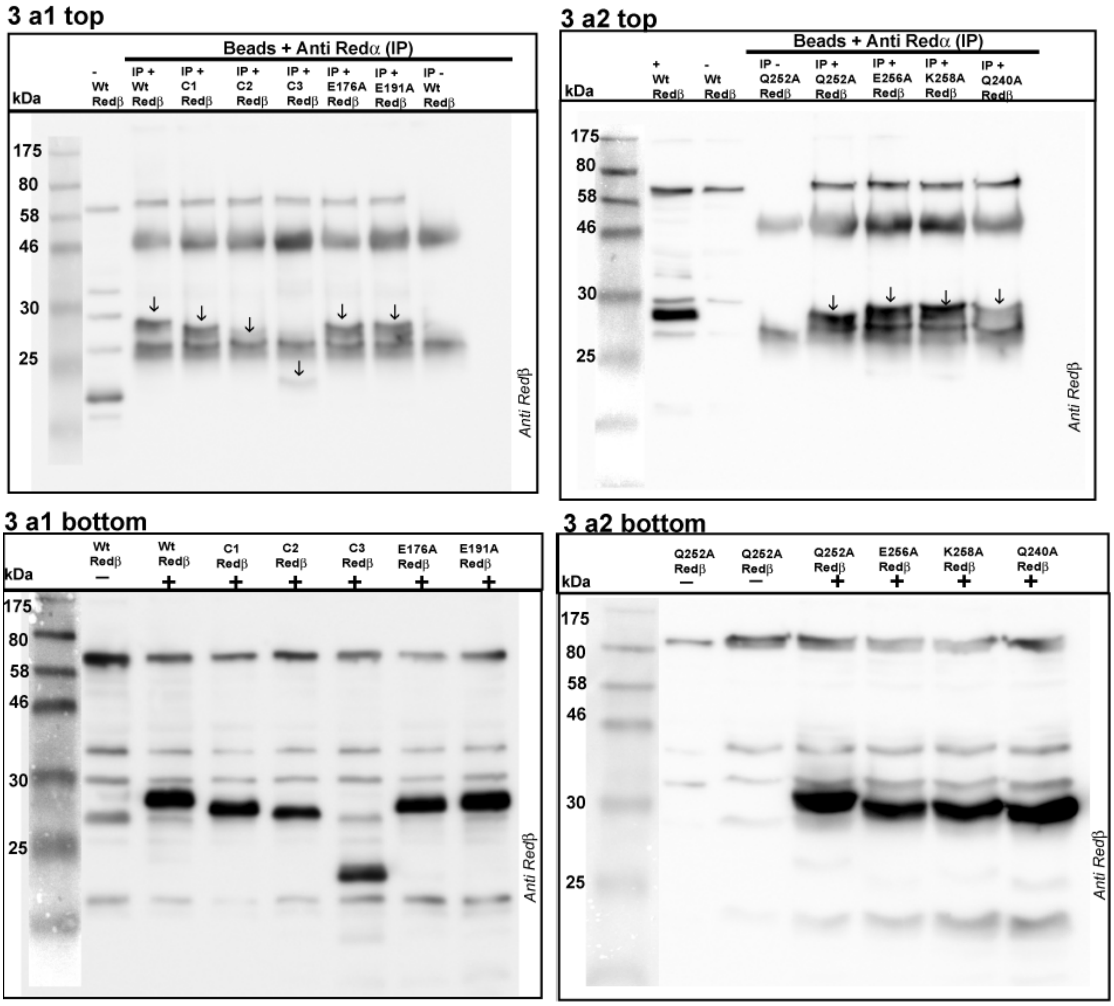

3 b top 1

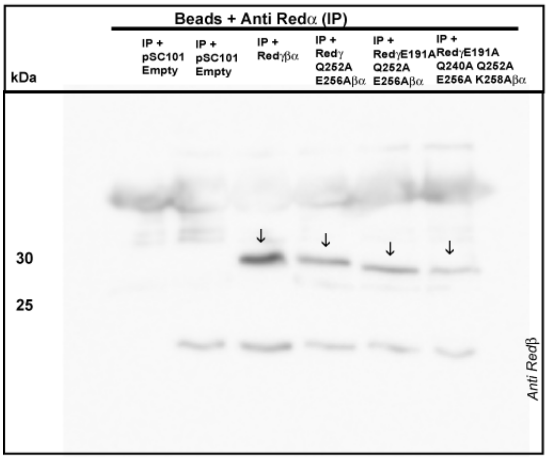

3 b top 2

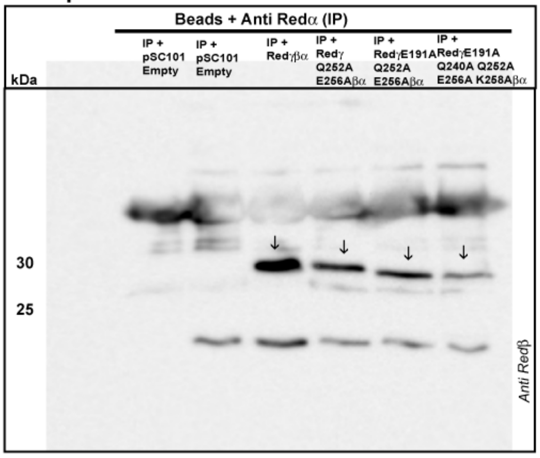

3 b bottom

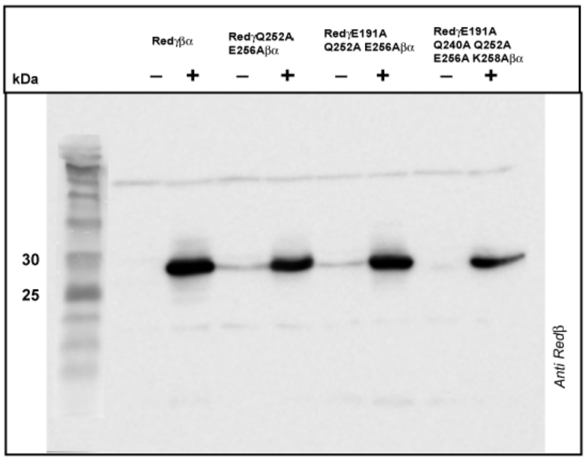

3 c top

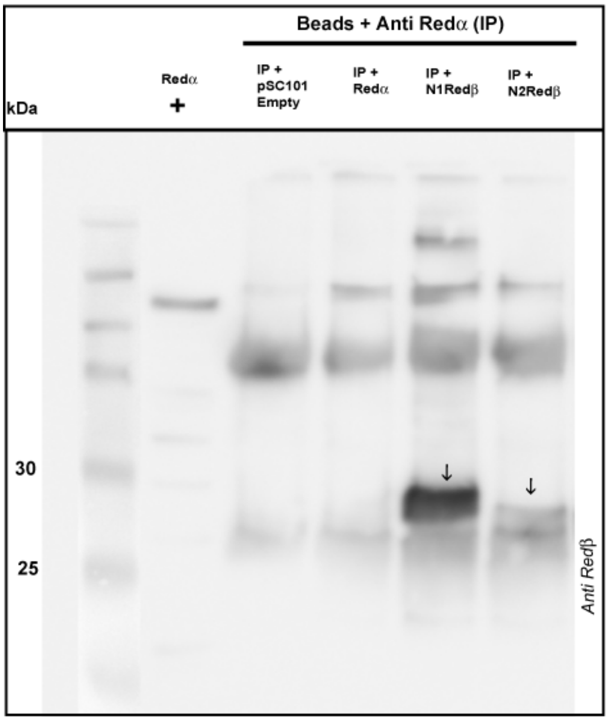

3 c bottom

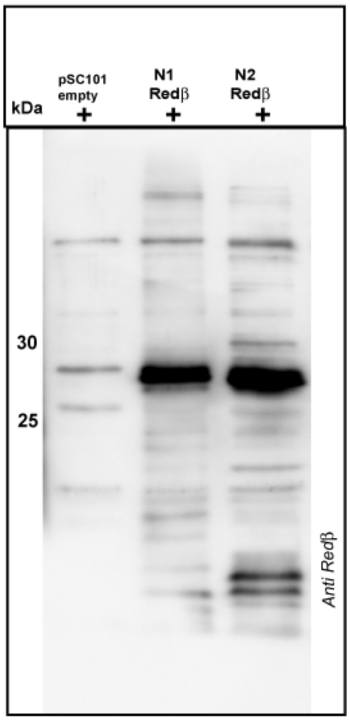

Supplementary Figure 4.

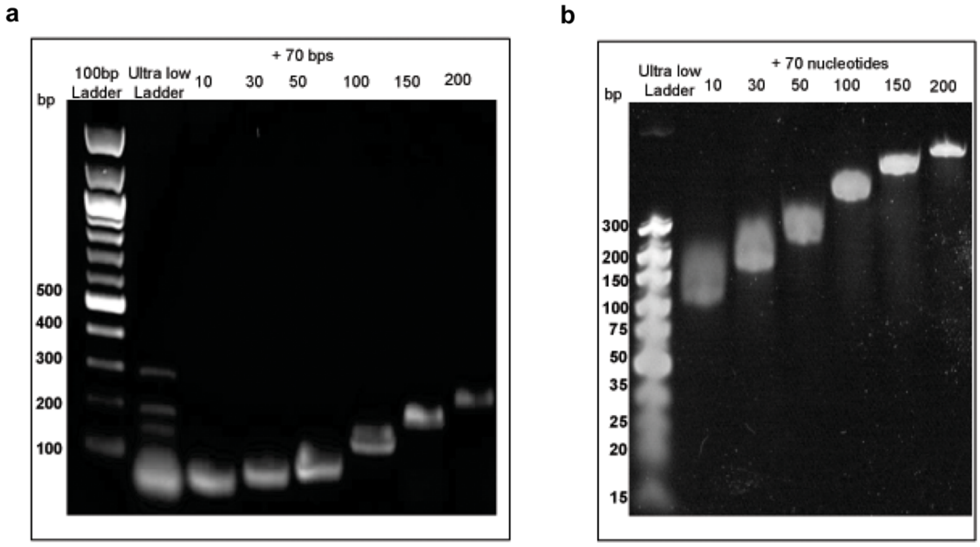

Supplementary Figure 5

DNA sequences used in Figure 2:

Size (nt)	50
Forward strand (5'-3')	CCATCCGCAAAAATCGAGCTATGCAGGGCGATTCTGCTCTAAGCCATCCG
Reverse strand (5'-3')	GCGGATGGCTTAGAGCAGAATCGCCCTGCATAGCTCGATTTTTGCGGATG

DNA sequences used in Figure S1c,d

Size (nt)	Forward strand (5'-3')	Reverse strand (5'-3')
10	GCTCTAAGCC	GGCTTAGAGC
14	GCTCTAAGCCATCC	GGATGGCTTAGAGC
15	GCTCTAAGCCATCCG	CGGATGGCTTAGAGC
16	GCTCTAAGCCATCCGC	GCGGATGGCTTAGAGC
17	GCTCTAAGCCATCCGCA	TGCGGATGGCTTAGAGC
18	GCTCTAAGCCATCCGCAA	TTGCGGATGGCTTAGAGC
19	GCTCTAAGCCATCCGCAAA	TTTGCGGATGGCTTAGAGC
20	GCTCTAAGCCATCCGCAAAA	TTTTGCGGATGGCTTAGAGC
22	GCTCTAAGCCATCCGCAAAAAT	ATTTTTGCGGATGGCTTAGAGC
